# Prophage-Derived Endolysin E1 Synergizes with Meropenem Against *Acinetobacter baumannii*

**DOI:** 10.3390/microorganisms14050953

**Published:** 2026-04-23

**Authors:** Jinyu Wang, Jinlong Bai, Yuhui Li, Ruirui Hu, Haihua Yang, Shengwei Hu, Wei Ni

**Affiliations:** College of Life Sciences, Shihezi University, Shihezi 832003, China; 20232006031@stu.shzu.edu.cn (J.W.);

**Keywords:** *Acinetobacter baumannii*, endolysin, genome, prophage, combination therapy

## Abstract

Carbapenem-resistant *Acinetobacter baumannii* (CRAB) is classified as a critical priority pathogen by the World Health Organization, and new therapeutic alternatives are urgently needed. In this study, we performed genomic mining of 27,531 *A. baumannii* genomes and identified 5144 prophage-derived endolysin candidates. Four highly prevalent candidates (E1–E4) were recombinantly expressed and functionally evaluated against *A. baumannii*. Among them, E1 exhibited the strongest bactericidal activity against reference strains ATCC 19606 and CMCC 25001, with a minimum inhibitory concentration in the micromolar range. E1 effectively disrupted preformed biofilms (>60% reduction) and remained stable under a broad range of temperatures (4–60 °C), pH values (6–8), and NaCl concentrations (up to 500 mM). Structural analysis indicated that E1 adopts a canonical lysozyme-like fold with key residues for peptidoglycan binding, and its lytic activity in vitro relied on 1 mM EDTA-mediated outer membrane permeabilization. In a murine peritoneal infection model, combination therapy with E1 and meropenem (each at 1 × MIC) significantly increased the survival rate to 66.7% and reduced bacterial loads in blood and multiple organs. This study demonstrates that prophage-derived endolysin E1 acts synergistically with meropenem against *A. baumannii*, supporting E1 as a promising candidate for developing combination therapies against CRAB.

## 1. Introduction

*Acinetobacter baumannii* has been widely recognized as a clinically significant multidrug-resistant Gram-negative pathogen in the 21st century. Hospital-acquired infections caused by this organism are increasingly prevalent worldwide, exhibiting a highly complex and continuously evolving epidemiological profile that poses a substantial challenge to current anti-infective therapeutic strategies [1]. According to the WHO Bacterial Priority Pathogens List (BPPL) 2024, updated by the World Health Organization (WHO) in 2024, 15 drug-resistant bacterial pathogens are categorized into three priority tiers—“critical,” “high,” and “medium”—based on their threat to public health. Among them, carbapenem-resistant *A. baumannii* (CRAB) is ranked at the top of the “critical priority” tier, underscoring the urgency and importance of developing novel antimicrobial agents and infection control measures against this pathogen [2,3,4]. Consequently, there is an imperative need to develop new antimicrobial therapeutics targeting CRAB.

Prophages denote temperate bacteriophage genomes that persist either as integrated elements within the bacterial chromosome or as extrachromosomal plasmids in the host cell. Serving as pivotal vectors of horizontal gene transfer, prophages play a substantial role in modulating virulence factor expression, mediating the dissemination of antimicrobial resistance genes, and driving genomic plasticity and evolutionary adaptation in bacteria [5]. In recent years, with deeper elucidation of prophage functionality, it has been consistently observed that their genomes frequently harbor genes encoding endolysins [6,7,8]. Endolysins constitute a class of phage-encoded peptidoglycan hydrolases that specifically degrade the peptidoglycan layer of the bacterial cell wall during the terminal phase of the phage lytic cycle, thereby affecting host cell lysis [9]. Owing to their exceptional target specificity and low propensity to induce resistance, endolysins exhibit distinctive advantages in anti-infective therapy: they enable precise eradication of pathogenic bacteria while minimally perturbing the commensal microbial community [10]. Currently, endolysins are being investigated across diverse administration routes—including parenteral, topical, and oral formulations—and demonstrate considerable promise in combating infections caused by multidrug-resistant Gram-positive and select Gram-negative pathogens [11,12,13].

Bacteriophages (phages) represent the most abundant viruses on Earth and are capable of specifically infecting and lysing their bacterial hosts [14]. Phages have long been recognized for their capacity to combat multidrug-resistant pathogens and biofilms [15]. Conventional approaches for phage isolation and cultivation are fraught with limitations, which have substantially impeded the development of phage-based therapeutics against multidrug-resistant Gram-negative bacteria such as *A. baumannii* [16,17,18]. The outer membrane barrier of Gram-negative bacteria hinders efficient phage adsorption and infection, resulting in low isolation yields and prolonged screening timelines [19]. In contrast, the strategy of directly mining and heterologously expressing endolysins from prophage genomes offers distinct advantages, providing a more efficient and safer avenue for the development of novel antimicrobial agents targeting *A. baumannii* [20,21,22].

The drug resistance of *A. baumannii* has been increasingly severe, and carbapenem-resistant *A. baumannii* (CRAB) has been listed as a pathogen of critical priority by the World Health Organization (WHO), with limited available therapeutic approaches. Prophage-encoded lysins possess the advantages of high target specificity and low propensity to induce drug resistance. However, the traditional phage isolation and culture methods suffer from drawbacks such as low isolation efficiency and long screening cycles, which fail to meet the demand for the research and development of anti-CRAB agents. Therefore, this study adopted a genomic mining strategy to screen novel lysins from prophages of *A. baumannii*, aiming to obtain candidate molecule E1 with excellent antibacterial activity and synergistic effects with clinical antibiotics, so as to address the therapeutic dilemma of CRAB.

## 2. Materials and Methods

### 2.1. Bacterial Genomes and Bioinformatic Analyses

A total of 27,531 *Acinetobacter baumannii* genomes were downloaded from the National Center for Biotechnology Information (NCBI). Prophage regions within these bacterial genomes were annotated using the bioinformatics tool VIBRANT 2.0 [23]; Subsequently, CheckV 1.0.3 [24] was employed to assess and filter out potential host-derived sequences from the prophage annotations; Finally, HMMER 3.0 [25] was utilized to perform sequence homology searches against the curated prophage dataset to identify endolysin-encoding sequences. The HMMER search was conducted using a custom hidden Markov model profile built from 293 experimentally validated endolysin amino acid sequences previously identified in our laboratory.

The three-dimensional structures of candidate endolysins were predicted using AlphaFold2, an artificial intelligence-based protein structure prediction tool. Structural visualization and analysis were performed using PyMOL 3.0 (Schrödinger, LLC, Framingham, MA, USA), a molecular graphics system for structural display and annotation.

### 2.2. Bacterial Strains and Culture Conditions

ATCC and CMCC strains were obtained from the Shanghai Microbial Culture Collection (SHMCC, Shanghai, China; https://www.shmcc.org.cn/, accessed on 28 April 2024). S and K strains were derived from clinical and environmental isolates previously archived in our laboratory. All strains were routinely cultivated in Luria–Bertani (LB) broth or brain–heart infusion (BHI) medium at 37 °C.

### 2.3. Molecular Cloning

The four endolysins selected for experimental validation were designated E1, E2, E3, and E4, respectively. Gene synthesis, prokaryotic expression, and protein purification were carried out by Zhongding Biotechnology (Nanjing, China). The coding sequences of E1–E4 were 594 bp, 678 bp, 576 bp, and 468 bp in length, respectively. Each endolysin gene fragment was seamlessly cloned upstream of the XhoI restriction site in the expression vector, and an N-terminal SUMO tag was incorporated to enhance the solubility of the recombinant protein.

### 2.4. Protein Expression and Purification

The recombinant expression plasmids constructed in this study were transformed into *Escherichia coli* ArcticExpress (DE3) competent cells (strain preserved by Zoonbio Biotechnology Co., Ltd., Nanjing, China). The transformation and expression procedures were performed as follows: 1 μL of plasmid DNA was added to 100 μL of BL21 (DE3) PlySSTM competent cells and incubated on ice for 30 min. The mixture was then subjected to heat shock at 42 °C for 90 s, immediately transferred back to ice for 5 min, and subsequently mixed with 600 μL of antibiotic-free LB broth. The cells were recovered by shaking at 37 °C and 200 rpm for 40 min. Following recovery, the culture was centrifuged, and the entire pellet was plated onto LB agar plates supplemented with 50 μg/mL ampicillin (Amp) or kanamycin (Kan), followed by overnight incubation at 37 °C in an inverted position.

On the following day, a single colony was inoculated into 3 mL of LB broth containing 50 μg/mL Amp and cultured overnight at 37 °C with shaking at 200 rpm. The next day, the culture was diluted 1:100 into 30 mL of fresh LB medium containing the appropriate antibiotic and grown at 37 °C and 200 rpm until the optical density at 600 nm (OD_600_) reached 0.6–0.8. One milliliter of culture was collected as an uninduced control, centrifuged at 10,000 rpm for 2 min at room temperature, and the supernatant was discarded. The cell pellet was resuspended in 100 μL of 1× SDS-PAGE loading buffer.

The remaining culture was divided into two groups for induction: (i) IPTG (isopropyl β-D-1-thiogalactopyranoside; Sigma-Aldrich, Saint Louis, MO, USA) was added to a final concentration of 0.2 mM, followed by induction at 37 °C and 200 rpm for 4 h; (ii) another aliquot received 0.2 mM IPTG and was induced overnight at 15 °C and 220 rpm to optimize soluble protein expression. After induction, 1 mL of each culture was harvested and processed as whole-cell lysates using the same method as the uninduced control. The remaining cultures were centrifuged at 4000 rpm for 10 min at 4 °C, and the pellets were resuspended in ice-cold PBS for sonication (parameters: 200 W, 2 s on/3s off, total duration 10 min, performed on ice). The lysates were then centrifuged at 12,000 rpm for 15 min to separate the soluble fraction (supernatant) from the insoluble fraction (pellet, containing inclusion bodies). Both fractions were resuspended in 1× loading buffer and analyzed by 12% SDS-PAGE, followed by Coomassie Brilliant Blue staining.

Purification of the target protein was carried out using Ni^2+^-IDA affinity chromatography. The soluble supernatant was loaded onto a Ni-IDA-Sepharose CL-6B affinity column (GE Healthcare) pre-equilibrated with Ni-IDA Binding Buffer (20 mM Tris–HCl, 150 mM NaCl, pH 8.0) at a flow rate of 0.5 mL/min. After loading, the column was washed with Binding Buffer at 0.5 mL/min until the OD_280_ of the effluent reached a stable baseline. Non-specifically bound proteins were removed by washing with Washing Buffer (20 mM Tris–HCl, 30 mM imidazole, 150 mM NaCl, pH 8.0) at 1 mL/min. The target protein was then eluted with Elution Buffer (20 mM Tris–HCl, 250 mM imidazole, 150 mM NaCl, pH 8.0) at 1 mL/min, and elution fractions were collected. The eluate was dialyzed overnight at 4 °C against PBS using a 10 kDa molecular weight cutoff dialysis membrane to remove imidazole. The purity and integrity of the final protein preparation were confirmed by 12% SDS-PAGE.

### 2.5. Antimicrobial Activity Assay

The concentrations of the purified endolysin proteins obtained from the aforementioned experiments were 1.7 mg/mL for E1, 0.5 mg/mL for E2, 1.0 mg/mL for E3, and 0.7 mg/mL for E4. The proteins were subsequently diluted with sterile PBS to the desired concentrations for different experiments.

To evaluate the antibacterial activity of the purified lysins, in vitro antibacterial assays were performed to examine the effects of E1, E2, E3, and E4 against *Acinetobacter baumannii* ATCC 19606 and CMCC 25001. The experimental groups were designed as follows: ① Blank control group (containing only LB medium); ② Bacterial control group (containing bacterial suspension without lysin); ③ Endolysin treatment groups (containing bacterial suspension plus different concentrations of endolysin proteins); ④ E1/E2/E3/E4 treatment groups (containing bacterial suspension plus the corresponding endolysin at their initial concentrations).

Briefly, the target strains were cultured to the mid-logarithmic phase (OD_600_ ≈ 0.5). The bacterial cells were collected by centrifugation, washed twice with sterile PBS (0.01 M, pH 7.4), and resuspended to a final concentration of approximately 1 × 10^6^ CFU/mL. A total of 100 μL of bacterial suspension was mixed with 100 μL of purified lysin solution (diluted in PBS), giving a final reaction volume of 200 μL. The final concentrations were set as follows: 850 μg/mL for E1, 250 μg/mL for E2, 500 μg/mL for E3, and 350 μg/mL for E4. Each concentration was tested in triplicate wells, and all experiments were independently repeated three times.

After incubation at 37 °C for 2 h, the mixture was transferred to 96-well flat-bottom microplates and continuously cultured at 37 °C for 16 h. The OD_600_ value was automatically recorded every 20 min to dynamically assess bacterial growth inhibition. Antibacterial activity was expressed as the relative inhibition rate, calculated using the following formula: Relative inhibition rate (%) = [OD_600_ (bacterial control) − OD_600_ (lysin-treated group)]/OD_600_ (bacterial control) × 100%.

To systematically evaluate the physicochemical stability of the endolysins, additional assays were conducted to assess tolerance to varying salt concentrations, temperatures, and pH conditions. For salt stability testing, the reaction buffer was prepared using 0.01 M PBS (pH 7.4) supplemented with NaCl at final concentrations of 0, 100, 200, 300, 400, or 500 mM; all other procedures remained unchanged. In thermal stability assays, purified endolysin was pre-incubated for 1 h at 4, 10, 20, 30, 37, 40, 50, 60, 70, or 80 °C, cooled to room temperature, and then mixed with *Acinetobacter baumannii* ATCC 19606 (1 × 10^6^ CFU/mL) in 0.01 M PBS. After 1 h of incubation at 37 °C, residual antimicrobial activity was determined. For pH stability assessment, endolysin was pre-incubated in buffers composed of 20 mM sodium phosphate and 150 mM NaCl, adjusted to pH values of 4.0, 5.0, 6.0, 7.0, 8.0, 9.0, 10.0, 11.0, or 12.0. Following pre-treatment, the enzyme was mixed with bacterial suspension and incubated at 37 °C for 1 h, after which the retained activity was evaluated.

Antimicrobial activity was expressed as the relative inhibition rate, calculated using the following formula: Relative Inhibition (%) = [OD600 (bacteria only) − OD600 (with endolysin)]/[OD600 (bacteria only)]. All experiments were performed in triplicate, independently repeated three times, to ensure reliability and reproducibility of the results.

### 2.6. Agar Diffusion Assay for Inhibition Zone Detection

The spot-on-lawn method was employed to assess the antibacterial activity of purified endolysin proteins against *Acinetobacter baumannii* ATCC 19606. The bacterial strain was cultured in LB broth at 37 °C with shaking until mid-logarithmic phase (OD_600_ ≈ 0.6). Cells were harvested by centrifugation at 6000× *g* for 5 min at 4 °C, washed two to three times with sterile phosphate-buffered saline (PBS) to completely remove residual LB broth, and then pelleted again under identical centrifugation conditions. For all in vitro assays against Gram-negative bacteria, outer membrane permeabilization was performed with 1 mM EDTA. The final cell pellet was resuspended in sterile PBS containing 1 mM EDTA and incubated at 37 °C for 15 min to permeabilize the outer membrane. Residual EDTA was removed by repeating the centrifugation and washing steps.

The bacterial suspension was then adjusted to a standardized concentration of 1 × 10^8^ colony-forming units (CFU)/mL using a nephelometer. A 200-μL aliquot of this suspension was evenly spread onto the surface of an LB agar plate and allowed to air-dry. Subsequently, 10 μL of endolysin protein solution was spotted onto the inoculated agar surface, with 10 μL of sterile PBS applied as a negative control. In this study, E1 was confirmed to possess antibacterial activity based on the results of antibacterial activity assays. To further investigate its inhibitory effects at different concentrations, E1 was diluted to 1700 μg/mL, 850 μg/mL, and 425 μg/mL. Plates were incubated at 37 °C for 16–18 h, after which the formation of inhibition zones was visually examined. Each experiment was performed in triplicate to ensure the reliability and reproducibility of the results.

### 2.7. Determination of Minimum Inhibitory Concentration

The minimum inhibitory concentration (MIC)—defined as the lowest concentration of endolysin that inhibits visible bacterial growth—was determined in Mueller–Hinton broth (MHB) using the broth microdilution method in accordance with guidelines from the Clinical and Laboratory Standards Institute (CLSI). In this study, the initial concentration of E1 used was 1700 μg/mL, which was mixed with an equal volume of bacterial suspension. The initial concentration of E1 used in this study was 1700 μg/mL. Following equal-volume mixing with bacterial suspension, it was serially diluted to 850 μg/mL, 425 μg/mL, 212.5 μg/mL, 106.25 μg/mL, 53.13 μg/mL, and 26.57 μg/mL. Exponentially growing bacterial cells were diluted to a final concentration of 1 × 10^6^ CFU/mL in Mueller–Hinton broth and dispensed into 96-well round-bottom microtiter plates. Serial dilutions of the purified endolysin protein were then added, and the plates were incubated at 37 °C for 16 h. The MIC was recorded as the lowest concentration at which no visible bacterial growth was observed. Each assay was performed in triplicate with independent biological replicates to ensure accuracy and reproducibility of the results.

### 2.8. Biofilm Eradication Assay

To evaluate the capacity of endolysin proteins to inhibit biofilm formation by *Acinetobacter baumannii*, 1 × MIC endolysin (10 μM) was introduced prior to biofilm maturation. Specifically, the protein was administered at two distinct stages of biofilm development: the early phase (12 h post-inoculation) and the mature phase (36 h post-inoculation). In this assay, biofilm formation was quantified using the crystal violet staining method in a 96-well microtiter plate-based biofilm assay. The extent of biofilm reduction mediated by the endolysin was determined by measuring the absorbance at OD_600_ following crystal violet extraction.

### 2.9. Lytic Spectrum Assessment

To evaluate whether endolysin proteins derived from *Acinetobacter baumannii* prophages exhibit broad-spectrum bactericidal activity, a panel of bacterial strains was employed, including 17 isolates of *Staphylococcus aureus*, 15 isolates of *Klebsiella pneumoniae*, 2 isolates of *Citrobacter* spp., 1 isolate of *Escherichia fergusonii*, and 1 strain of *Pseudomonas aeruginosa* PAO1. The experimental procedure was performed in accordance with the previously described antimicrobial activity assay.

### 2.10. Murine Infection Model

All animal experiments were approved by the Animal Ethics Committee of Shihezi University, China. Six-week-old female SPF BALB/c mice were used in this study. First, the median lethal dose (LD_50_) of *A. baumannii* ATCC 19606 in mice was determined. Mice were housed 4 per cage and injected with concentrations of 5 × 10^6^, 5 × 10^7^, 5 × 10^8^, and 5 × 10^9^ CFU per mouse, respectively. Mortality was observed 24 h post-infection. All mice in the 5 × 10^6^ CFU group survived; 2 mice survived in the 5 × 10^7^ CFU group; and all mice died in the 5 × 10^8^ and 5 × 10^9^ CFU groups. Thus, the LD_50_ was determined to be 5 × 10^7^ CFU per mouse. In subsequent therapeutic experiments, the challenge dose was set at 2 × LD_50_, which was approximately 1 × 10^8^ CFU per mouse as confirmed by viable cell counting.

Mice were randomly numbered and divided into 6 groups (n = 6 per group): ① Normal control group (uninfected, intraperitoneal injection of 200 μL sterile PBS); ② Model control group (infected with *A. baumannii*); ③ Meropenem monotherapy group (infected mice treated with 200 μL meropenem at 2 × MIC); ④ E1 + meropenem combination group (infected mice treated with 200 μL E1 at 1 × MIC plus meropenem at 1 × MIC).

All mice were acclimated for 3 days before experiments under consistent sterile conditions. Drinking water, bedding, and feed were autoclaved. The housing environment was maintained at 22 ± 2 °C, 50 ± 5% humidity, and a 12 h/12 h light–dark cycle.

Mice were challenged with *A. baumannii* ATCC 19606 at the predetermined LD_50_-based dose. One hour after infection, endolysin and antibiotic were administered intraperitoneally as single-dose treatments, with an injection volume of 200 μL per mouse. For bacterial suspension preparation: *A. baumannii* ATCC 19606 was cultured to mid-log phase (OD_600_ ≈ 0.5). Bacteria were harvested by centrifugation at 6000× *g*, 4 °C for 5 min, washed twice with sterile PBS, and resuspended to 5 × 10^9^ CFU/mL for intraperitoneal infection at 200 μL per mouse (1 × 10^8^ CFU per mouse).

The previously determined MIC of endolysin E1 against strain ATCC 19606 was used as the therapeutic dose. Meropenem was weighed and dissolved in PBS to prepare the antibiotic solution. Survival rates within 48 h were analyzed using the Kaplan–Meier method and log-rank test. For bacterial burden assays, mice were euthanized, and liver, spleen, kidney, lung, and blood samples were collected under sterile conditions. Organ samples were weighed and homogenized at 2000 rpm for 30 s on ice with sterile PBS at a ratio of 1:10 (*w*/*v*). Blood and organ homogenates were serially diluted from 10 to 10^6^ with sterile PBS. Aliquots of 100 μL were spread onto LB agar plates and incubated at 37 °C for 16–18 h. Colonies were counted to calculate bacterial loads in organs (CFU/g) and blood (CFU/mL).

During the experiment, moribund mice (lethargy, inability to eat or drink, body weight loss ≥ 20%) were humanely euthanized by cervical dislocation. Surviving mice were also euthanized by cervical dislocation at the end of the experiment. All procedures were performed in accordance with the guidelines of the Animal Ethics Committee of Shihezi University (Ethics Approval No.: A2025-937). EDTA was only used for in vitro outer membrane permeabilization and was not administered to mice in any in vivo experiments.

### 2.11. Statistical Analysis

All experimental data were statistically analyzed using SPSS 26.0 software. Quantitative data were expressed as mean ± standard deviation (x ± s). Comparisons between groups were performed using one-way analysis of variance (ANOVA) for multiple groups (e.g., among different lysin treatment groups, different E1 concentration groups) or Student’s *t*-test for two groups (e.g., E1 alone group vs. E1 + EDTA group, combination therapy group vs. monotherapy group). Survival rates of mice were analyzed using the Kaplan–Meier method and log-rank (Mantel–Cox) test. A value of *p* < 0.05 was considered statistically significant, and *p* < 0.01 was considered highly statistically significant.

## 3. Results

### 3.1. Identification of Endolysins from Acinetobacter baumannii and Its Prophage Genomes

**Figure 1:**
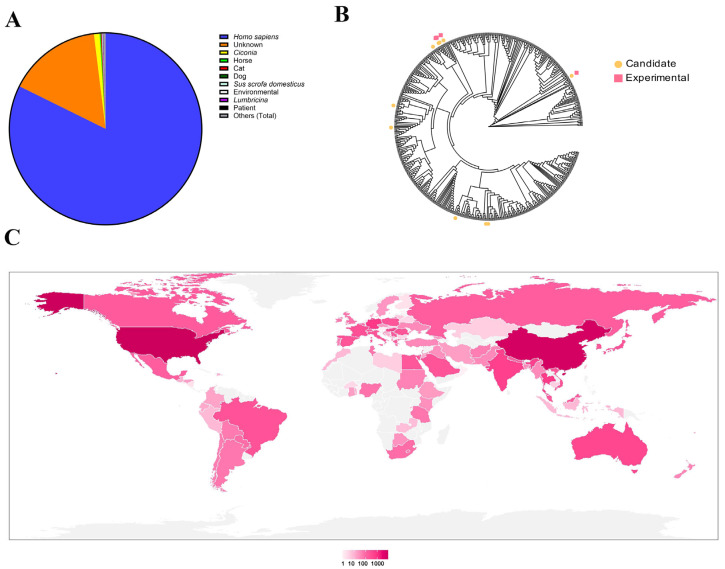
Overview of *Acinetobacter baumannii*-related genomic data. (**A**) Host origin distribution of 27,531 *A. baumannii* genomes. The pie chart indicates that the vast majority were isolated from human clinical specimens, with minor contributions from animal and environmental sources. (**B**) Phylogenetic tree of *A. baumannii* prophages, annotated with frequently occurring endolysins (yellow: “candidate” endolysins; pink: sequences associated with experimentally validated endolysins), illustrating the diversity and abundance of endolysins across distinct strains. (**C**) Global geographic distribution of *A. baumannii* isolates. The world map uses color intensity to represent the number of genome submissions per country, underscoring the pathogen’s extensive worldwide dissemination.

Initial analysis of 27,531 *Acinetobacter baumannii* genomes revealed that the majority were isolated from human clinical specimens, with a smaller proportion originating from animal sources (Figure 1A). These isolates exhibited a global distribution, spanning most countries worldwide (Figure 1C). Bioinformatic analysis of the 27,531 *A. baumannii* genomes downloaded from NCBI was performed using the VIBRANT pipeline, which annotated a total of 149,315 putative prophages. Subsequent quality assessment with CheckV enabled the removal of host-derived genomic fragments and low-quality sequences, resulting in a refined dataset of 93,587 high-confidence prophages.

To identify endolysin-encoding sequences within these prophages, HMMER—based homology searches were conducted using a custom profile derived from 293 experimentally validated endolysin amino acid sequences previously characterized in our laboratory. This analysis yielded 5144 distinct endolysin sequences, detected cumulatively across 134,104 genomic loci. Clustering and frequency analysis identified 10 endolysins exhibiting both high occurrence rates and broad host coverage.

**Figure 2:**
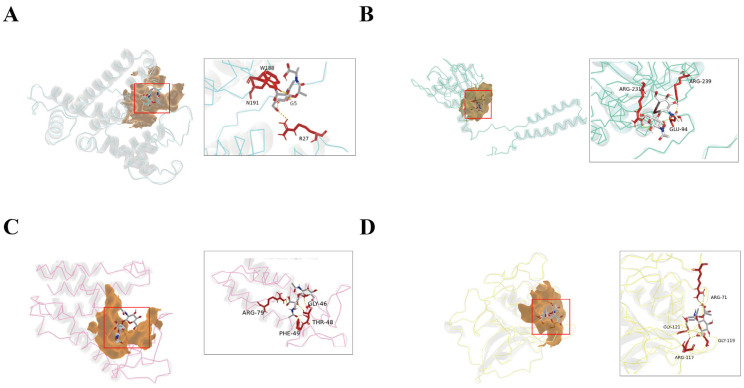
Predicted peptidoglycan-binding sites of candidate endolysins. (**A**) E1. (**B**) E2. (**C**) E3. (**D**) E4. Putative cleavage sites of each candidate endolysin were predicted using distinct peptidoglycan fragments as ligands. The three-dimensional structures of the endolysins were modeled using AlphaFold2 [26]. Amino acid residues (shown in red) involved in binding to the N-acetylmuramic acid (NAM)–N-acetylglucosamine (NAG) disaccharide ligand (shown in blue) were identified and visualized using PyMOL 3.0 [27]. NAM: N-acetylmuramic acid; NAG: N-acetylglucosamine.

**Figure 3:**
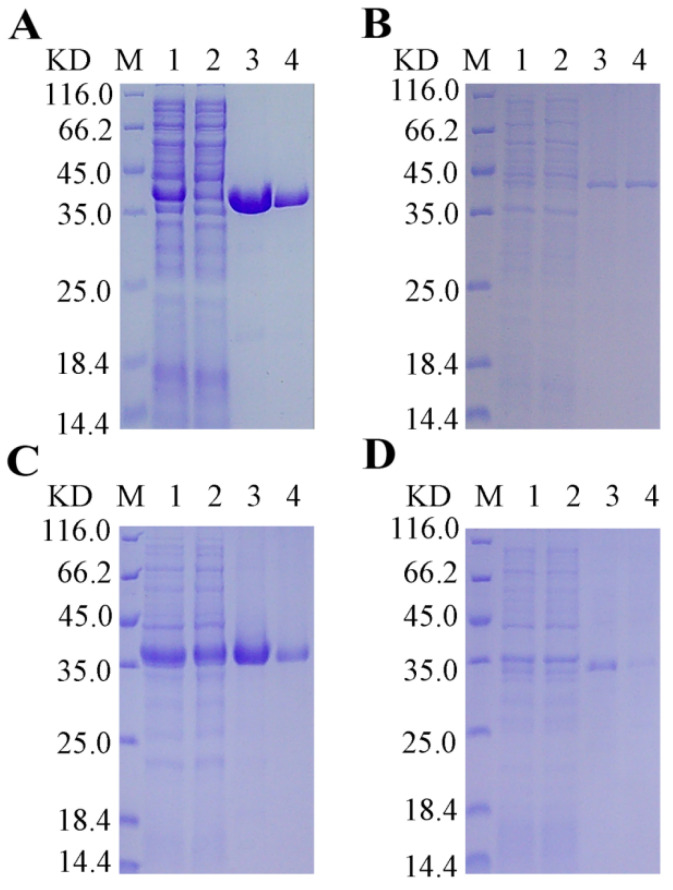
SDS-PAGE analysis of purified endolysins E1, E2, E3, and E4. (**A**) SDS-PAGE analysis of recombinant E1. M denotes the protein molecular weight marker; lane 1, post-lysis crude lysate; lane 2, flow-through fraction; lanes 3 and 4, elution fractions. The predicted molecular mass is 35.99 kDa. (**B**) SDS-PAGE analysis of recombinant E2. M denotes the protein molecular weight marker; lane 1, post-lysis crude lysate; lane 2, flow-through fraction; lanes 3 and 4, elution fractions. The predicted molecular mass is 39.16 kDa. (**C**) SDS-PAGE analysis of recombinant E3. M denotes the protein molecular weight marker; lane 1, post-lysis crude lysate; lane 2, flow-through fraction; lanes 3 and 4, elution fractions. The predicted molecular mass is 34.87 kDa. (**D**) SDS-PAGE analysis of recombinant E4. M denotes the protein molecular weight marker; lane 1, post-lysis crude lysate; lane 2, flow-through fraction; lanes 3 and 4, elution fractions. The predicted molecular mass is 31.37 kDa.

Based on these criteria, four representative endolysins—designated E1, E2, E3, and E4—were selected for functional validation (Figure 1B and Figure 2). Structural analysis of these candidates revealed the presence of α-helical domains, a hallmark feature of the lysozyme family. The four endolysin proteins were successfully expressed and purified (Figure 3).

### 3.2. In Vitro Antimicrobial Efficacy of Endolysin Proteins

To evaluate the bacteriostatic activities of E1, E2, E3, and E4 proteins, *A. baumannii* ATCC 19606 and CMCC 25001 were used as test strains. Strains were cultured to the logarithmic growth phase, incubated with each protein in phosphate-buffered saline (PBS) at 37 °C for 16 h, and the OD_600_ value was measured every 20 min. The results showed that the relative inhibition rate of E1 reached 89.2% ± 3.5% against *A. baumannii* ATCC 19606 and 87.6% ± 4.1% against CMCC 25001, which was significantly higher than those in the E2, E3, and E4 treatment groups, as well as the bacterial control group (*p* < 0.01).

**Figure 4:**
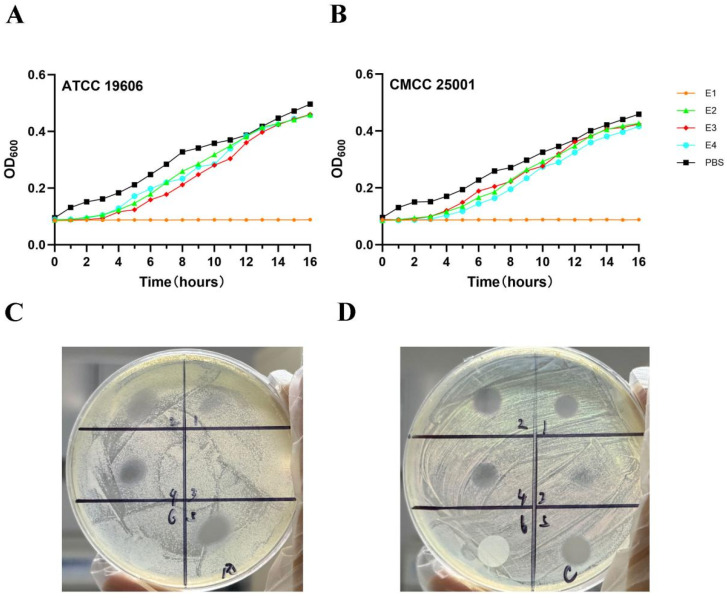
In vitro validation of the antibacterial activity of endolysin proteins. (**A**) Antibacterial efficacy of different endolysins against *Acinetobacter baumannii* ATCC 19606. (**B**) Antibacterial efficacy of different endolysins against *A. baumannii* CMCC 25001. (**C**) Agar diffusion assay assessing endolysin activity against *A. baumannii* ATCC 19606: spots 1, 2, and 4 received E1 at concentrations of 4.25 μg, 8.5 μg, and 17 μg, respectively; spot 3 received 1 mM EDTA; spot 5 contained the lowest concentration of E1 (4.25 μg) combined with EDTA; spot 6 was loaded with PBS as a negative control. (**D**) Agar diffusion assay using *A. baumannii* CMCC 25001 as the test strain, with identical treatment conditions as in panel (**C**).

After E1 treatment, the OD_600_ values of both ATCC 19606 and CMCC 25001 remained below 0.1, whereas the OD_600_ values in the E2, E3, and E4 groups were all above 0.3, with no significant difference compared with the bacterial control group (*p* > 0.05). The bacteriostatic activity of E1 against the two strains was significantly higher than that of the other three proteins, indicating that E1 exerted a remarkable inhibitory effect (Figure 4A,B).

To further explore the antibacterial mechanism of E1, the two aforementioned strains were used as models to investigate the effect of EDTA (an outer membrane permeabilizer) on the activity of E1 (Figure 4C,D). Agar diffusion assays showed that the inhibition zone diameter was 18.6 ± 1.2 mm in the group treated with 17 μg E1 alone, and 22.3 ± 1.5 mm in the group treated with 4.25 μg E1 combined with EDTA, which was significantly larger than that of the E1 alone group (*p* < 0.05). In contrast, no obvious inhibition zone was observed in the EDTA alone group (diameter < 6 mm), demonstrating that EDTA could significantly enhance the antibacterial activity of E1.

### 3.3. Endolysin Sensitivity

**Figure 5:**
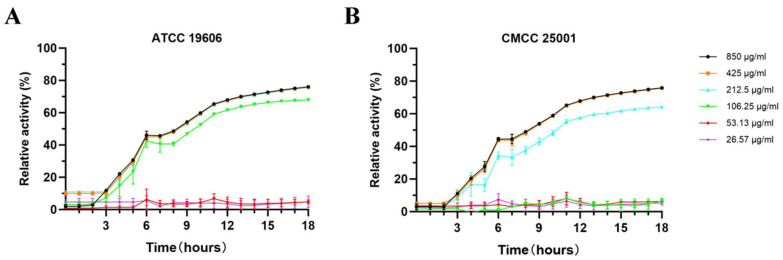
Minimum inhibitory concentration (MIC) of endolysin proteins. (**A**) MIC of endolysin E1 against *Acinetobacter baumannii* ATCC 19606. (**B**) MIC of endolysin E1 against *A. baumannii* CMCC 25001.

The minimum inhibitory concentrations (MICs) of E1 against *Acinetobacter baumannii* ATCC 19606 and CMCC 25001 were determined using the aforementioned experimental methods (Figure 5A,B).

### 3.4. Biofilm Disruption by Endolysin Proteins

**Figure 6:**
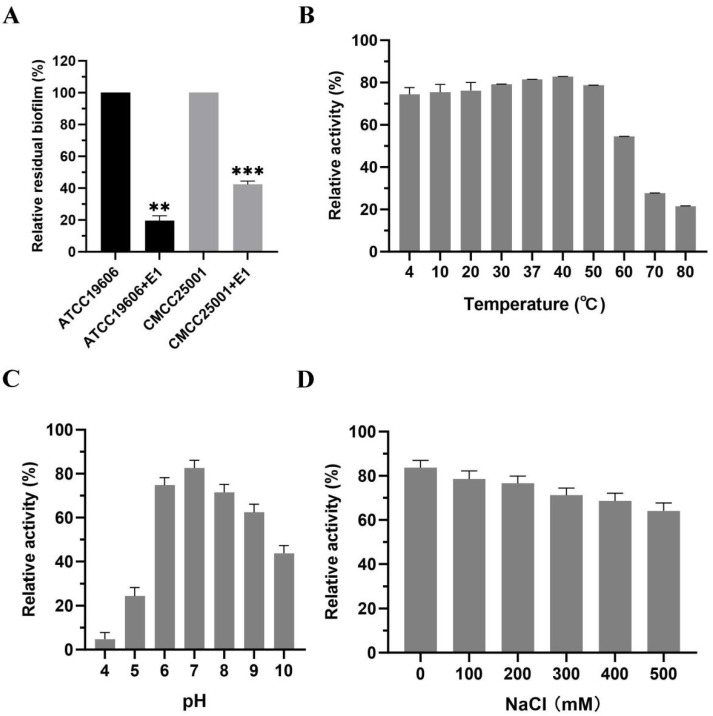
Biofilm disruption and biological characteristics of the endolysin protein. (**A**) Biofilm degradation efficacy. (**B**) Effect of temperature. (**C**) pH. (**D**) NaCl concentration on the lytic activity of endolysin E1. The test strain used was *Acinetobacter baumannii* ATCC 19606. In all assays, the maximum activity of E1 under the tested conditions was normalized to 100% to facilitate comparison. Each experiment was performed in triplicate. Error bars represent standard deviations. Statistical significance was determined by one-way ANOVA followed by Dunnett’s multiple comparisons test. ** *p* < 0.01 and *** *p* < 0.001 versus the corresponding untreated control.

Using the aforementioned experimental methodology, the results demonstrated that E1 exhibited a pronounced biofilm-reducing effect, achieving biofilm reduction rates exceeding 60% for both bacterial strains (Figure 6A).

### 3.5. Biological Characteristics of the Endolysin Protein

Using *Acinetobacter baumannii* strain ATCC 19606 as the target substrate, the effects of temperature, pH, and salinity (NaCl concentration) on the activity of E1 were evaluated. E1 exhibited a broad thermal stability profile, retaining activity across a temperature range of 4 °C to 60 °C (Figure 6B). Within the tested pH range of 4 to 10, E1 demonstrated relatively high lytic activity at pH 6–8 (Figure 6C). The optimal pH identified in this assay was subsequently used to assess the influence of NaCl concentration on E1 activity. Notably, E1 retained approximately 70% of its relative activity against *A. baumannii* ATCC 19606 even at an NaCl concentration as high as 500 mM (Figure 6D).

### 3.6. Lytic Spectrum Activity Analysis

**Figure 7:**
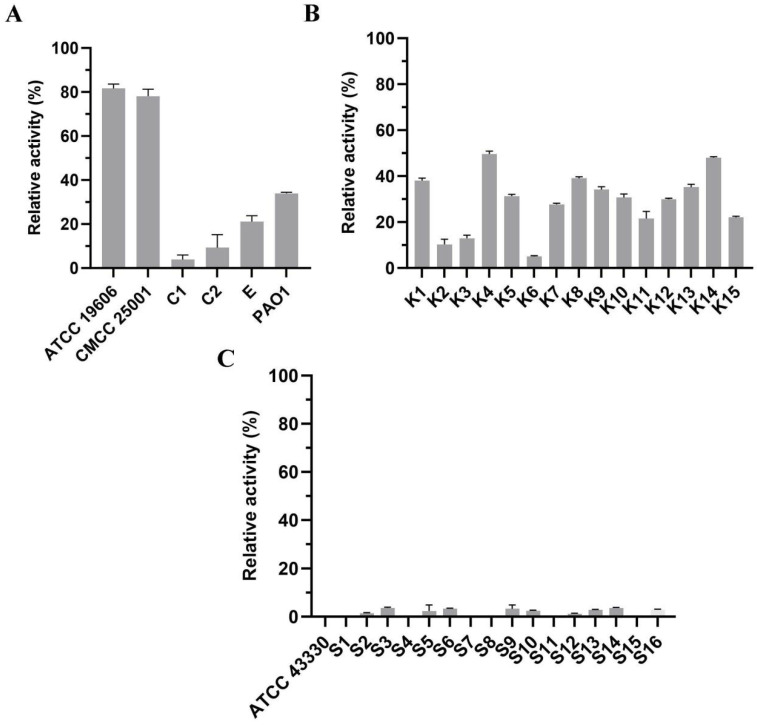
Lytic spectrum of endolysin E1 against representative bacterial strains. (**A**) Lytic activity against Gram-negative strains including *Acinetobacter baumannii*, *Citrobacter* spp., *Escherichia coli*, and *Pseudomonas aeruginosa*. (**B**) Lytic activity against *Klebsiella pneumoniae* isolates. (**C**) Lytic activity against Gram-positive *Staphylococcus aureus* isolates. All assays were performed in triplicate, and data are shown as mean ± standard deviation.

Due to the protective barrier conferred by the outer membrane (OM) of Gram-negative bacteria, E1 is unable to directly lyse these organisms. Therefore, prior to E1 treatment, all tested Gram-negative strains were pretreated with 1 mM EDTA at 37 °C for 15 min to permeabilize the OM. We observed that E1 exhibited lytic activity against the majority of the tested Gram-negative strains; however, the extent of lysis was relatively modest (Figure 7A,B). In contrast, E1 displayed no detectable lytic activity against the Gram-positive bacterium *Staphylococcus aureus* (Figure 7C).

### 3.7. Combination Therapy with Endolysin and Antibiotic in a Murine Model of Acinetobacter baumannii Infection

To evaluate the therapeutic efficacy of the endolysin protein, mice in the treatment groups were administered 200 μL of meropenem at 2 × MIC, or 200 μL of a mixture of 1 × MIC E1 plus 1 × MIC meropenem, at 0.5 h post-challenge with *A. baumannii* ATCC 19606. Mice in the other groups received an equal volume of PBS.

**Figure 8:**
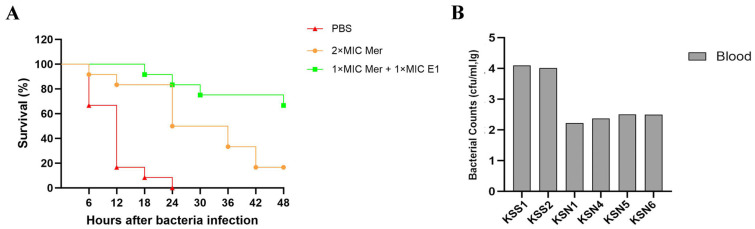
In vivo therapeutic efficacy of endolysin E1 combined with meropenem in a murine peritoneal infection model. (**A**) Survival curves of mice within 48 h (*n* = 6 per group). (**B**) Bacterial loads in mouse blood. Groups: Control, uninfected normal mice; Model, infected mice without treatment; KSS, infected mice treated with meropenem alone; E1 + KSN, infected mice treated with E1 plus meropenem. Abbreviation: cfu, colony-forming unit. Data are presented as mean ± standard deviation.

Mouse survival experiments showed that all mice in the model control group died within 48 h, with a survival rate of 0%. The survival rate was 16.7% in the meropenem monotherapy group and 66.7% in the E1 plus meropenem combination group (Figure 8A). The survival rate of the combination group was significantly higher than that of the monotherapy group (*p* < 0.01).

Bacterial burden assays revealed that bacterial loads in the liver, spleen, and blood of mice in the combination group were significantly lower than those in the monotherapy group (*p* < 0.01).

In a complementary bacterial burden study, organs were harvested and homogenized from mice treated with either meropenem alone or the meropenem—E1 combination, while organs from control mice were collected upon reaching a moribund state. Furthermore, in mice infected with *A. baumannii* ATCC 19606, the combination therapy led to a substantially greater reduction in blood bacterial counts compared to antibiotic treatment alone (Figure 8B).

**Figure 9:**
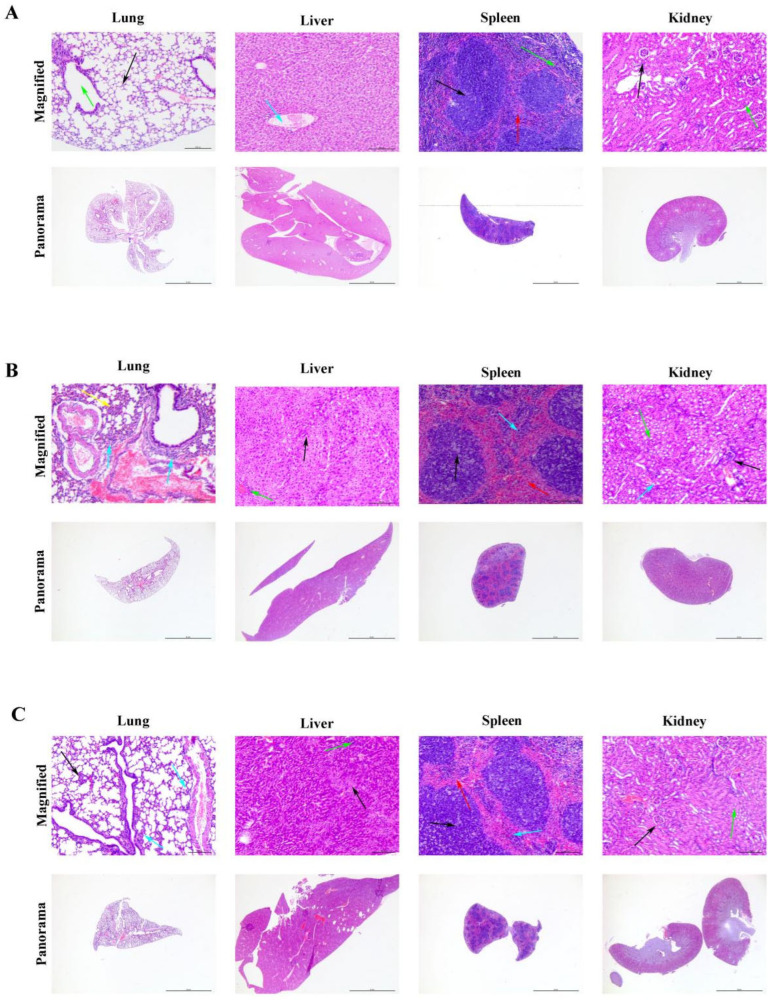
Histological sections of murine tissues. (**A**) Healthy control group: lung—alveolar architecture (black arrows), bronchioles (green arrows); liver—portal tracts (blue arrows); spleen—white pulp (black arrows), red pulp (red arrows), macrophages (green arrows); kidney—glomeruli (black arrows), renal tubules (green arrows). (**B**) Monotherapy group: lung—inflammatory cell infiltration (blue arrows), alveolar epithelial hyperplasia (yellow arrows); liver—focal hepatocellular edema (black arrows), mild inflammatory cell infiltration (green arrows); spleen—mild white pulp hyperplasia (black arrows), moderate red pulp congestion (red arrows), slight macrophage proliferation (blue arrows); kidney—tubular epithelial cell edema and degeneration (green arrows), glomeruli of variable size with partial atrophy (black arrows), diffuse interstitial edema and inflammatory cell infiltration (blue arrows). (**C**) Combination therapy group: lung—mild perivascular and peribronchial inflammatory infiltration (blue arrows), occasional minor inflammatory exudates in alveolar lumens (black arrows); liver—mild hydropic degeneration (black arrows), minimal inflammatory cell infiltration in portal tracts and hepatic lobules (green arrows); spleen—mild white pulp hyperplasia (black arrows), moderate red pulp congestion (red arrows), slight macrophage proliferation (blue arrows); kidney—renal tubules (green arrows), glomeruli (black arrows), with overall preserved architecture and negligible pathology.

Histopathological observations revealed significant differences in the degree of inflammation across multiple organs among the various experimental groups. Upon examination, healthy control mice (Figure 9A) showed normal tissue architecture, while those in the monotherapy group (Figure 9B) exhibited severe lung pathology characterized by widespread alveolar structural disruption, extensive infiltration of neutrophils within alveolar spaces, bronchial walls, and pulmonary interstitium, along with notable alveolar epithelial hyperplasia. In contrast, the combination therapy group (Figure 9C) displayed only mild inflammatory cell infiltration around blood vessels and bronchi, occasional minor exudates in alveolar spaces, and largely intact alveolar structures with minimal pathological changes, closely resembling the healthy control group.

In the liver, the monotherapy group showed disordered hepatic cords with focal hepatocyte edema and mild inflammatory infiltration in portal areas. However, the combination therapy group maintained well-preserved hepatic lobule structures, with only occasional mild hydropic degeneration and a negligible number of inflammatory cells, indicating significantly less pathological damage compared to both the infected and monotherapy groups.

Regarding the spleen, the monotherapy group demonstrated moderate immune activation, evidenced by slight white pulp hyperplasia, enlarged germinal centers, congested red pulp, and inflammatory cell infiltration. Notably, the combination therapy group also exhibited similar immune activation, suggesting that although the infection was effectively controlled, the immune system remained moderately activated.

In the kidneys, the monotherapy group displayed tubular epithelial cell edema and degeneration, partial glomerular atrophy, and interstitial edema with inflammatory cell infiltration. Conversely, the combination therapy group showed essentially normal glomerular and tubular structures, with only rare scattered inflammatory cells in the interstitium and no significant damage, highlighting the substantial protective effect of the combined treatment on renal tissue.

In summary, the combination of E1 with meropenem significantly mitigated multi-organ inflammatory responses, effectively maintaining tissue structural integrity. This therapeutic approach proved markedly superior to both untreated and monotherapy groups in terms of efficacy and tissue preservation.

## 4. Discussion

In this study, we systematically identified highly prevalent endolysin genes from *Acinetobacter baumannii* genomes using bioinformatic approaches and subsequently validated their in vitro antimicrobial activity and in vivo therapeutic efficacy. We found that the combination of endolysin E1 with meropenem significantly enhanced mouse survival and markedly reduced bacterial burdens in multiple organs, offering a promising combinatorial therapeutic strategy against multidrug-resistant *A. baumannii* infections [28,29,30]. This finding not only expands the applicability of endolysins in the treatment of Gram-negative bacterial infections [31], but also provides a novel avenue for addressing carbapenem-resistant *A. baumannii* (CRAB), a critical global public health threat [32].

The in vitro antimicrobial activity of endolysin E1 was markedly superior to that of other screened endolysins, a property closely associated with its distinctive molecular structural features. Three-dimensional structural modeling using AlphaFold2 revealed that E1 harbors a canonical α-helical architecture, consistent with the hallmark structural motif of the lysozyme family, which likely underpins its potent lytic activity [33,34]. Notably, in vitro pretreatment with 1 mM EDTA significantly enhanced the antibacterial efficacy of E1. EDTA was only used in vitro and not applied in vivo, in alignment with previously reported mechanisms by which endolysins act against Gram-negative bacteria [35,36,37]. As a metal chelator, EDTA disrupts the stability of the outer membrane by sequestering divalent cations essential for maintaining its integrity, thereby increasing membrane permeability. The outer membrane of *A. baumannii* is particularly dense, enriched with highly expressed outer membrane proteins and lipopolysaccharides that collectively constitute a formidable physical barrier, impeding direct access of endolysins to the underlying peptidoglycan layer [38]. Consequently, EDTA pretreatment appears to be a critical step in overcoming this barrier and enabling effective endolysin-mediated lysis.

The pronounced biofilm-disrupting efficacy of endolysin E1 (>60% reduction) underscores its potential clinical advantage. Biofilm formation is a hallmark of *A. baumannii* nosocomial infections, with the majority of clinically isolated multidrug-resistant strains reported to be capable of robust biofilm production [39]. Biofilm formation not only prolongs bacterial persistence in hospital environments but also enhances resistance to multiple antimicrobial agents through various mechanisms, including restricted antibiotic penetration and upregulation of efflux pump gene expression [40]. The biofilm-degrading activity of E1 may be attributed to its direct disruption of biofilm architecture or its potential to enhance EDTA-mediated penetration through the biofilm matrix. This property holds significant therapeutic relevance for the management of chronic infections associated with biofilms.

The synergistic effect observed between endolysin and antibiotic co-administration constitutes a key finding of this study. In the murine infection model, the survival rate in the group treated with the combination of E1 and meropenem (66.67%) was significantly higher than that in the group receiving meropenem alone (16.7%), suggesting that the endolysin may mitigate antimicrobial resistance risk by either directly compromising bacterial cell wall integrity or enabling reduced antibiotic dosing. This observation aligns with the general trend reported in the literature regarding the synergistic interactions between endolysins and conventional antibiotics. For instance, studies have demonstrated that the pneumococcal endolysin Cpl-711 exhibits synergistic activity when combined with cefotaxime or moxifloxacin against Streptococcus pneumoniae [41], and the staphylococcal endolysin CF301 acts synergistically with vancomycin or daptomycin against methicillin-resistant *Staphylococcus aureus* (MRSA) [42].

Nevertheless, this study has several limitations. First, the relatively small sample size in the in vivo murine experiments may compromise statistical power and the reproducibility of the findings. Second, systemic administration of EDTA in vivo may induce electrolyte imbalances and depletion of essential trace elements, necessitating further investigation into the safety of localized delivery or low-dose regimens [43]. Moreover, the intraperitoneal infection model using *A. baumannii* may not fully recapitulate the pathological microenvironment of clinically relevant infections, such as pneumonia or urinary tract infections [44]. Future studies should therefore employ infection models that more closely mimic human disease contexts to better evaluate therapeutic efficacy.

Against *A. baumannii*, a formidable multidrug-resistant pathogen, endolysins represent a promising class of novel antimicrobial agents with distinct advantages. Endolysins specifically target the peptidoglycan layer of the bacterial cell wall—a highly conserved structure that is not readily altered by bacteria—thereby minimizing the likelihood of resistance development [45]. Unlike conventional antibiotics, endolysins exhibit low cytotoxicity toward host tissues and exert minimal disruption on commensal microbial communities [46]. Furthermore, endolysins act instantaneously without the latent period associated with phage-mediated lysis [47], a characteristic of critical importance in the management of acute infections.

Future research should prioritize overcoming the current limitations of endolysin-based therapeutics against Gram-negative pathogens. The development of targeted delivery systems—such as lipid nanoparticles or cell-penetrating peptide–endolysin fusion constructs—may offer an effective strategy to circumvent EDTA dependency [48,49]. For instance, the amphipathic cell-penetrating peptide PEP-1, previously reported to bind the outer membrane via hydrophobic domains, may directly enhance endolysin penetration across the outer membrane [50]. Additionally, exploring synergistic combinations of endolysins with other antimicrobials—such as polymyxins [51] or tigecycline [52]—as well as engineering modified endolysins (e.g., E1 derivatives fused with membrane-translocating peptides) [53], could further augment therapeutic efficacy. Finally, comprehensive long-term toxicity assessments and studies employing infection models that more faithfully recapitulate clinical scenarios will be essential to facilitate the translational progression of endolysins from bench to bedside.

In summary, this study, through bioinformatic screening and functional validation, has, for the first time, demonstrated the synergistic therapeutic effect of endolysin E1 in combination with meropenem against *A. baumannii* infection, thereby providing a theoretical foundation and experimental evidence for the development of novel antimicrobial agents targeting carbapenem-resistant *A. baumannii* (CRAB). This finding not only broadens the applicability of endolysins in the treatment of Gram-negative bacterial infections but also offers a novel strategic perspective for addressing the global public health challenge posed by multidrug-resistant bacterial infections.

## Data Availability

The data presented in this study are available on request from the corresponding author due to ethical and privacy restrictions.
